# Safety of bridging antiplatelet therapy with the gpIIb-IIIa inhibitor tirofiban after emergency stenting in stroke

**DOI:** 10.1371/journal.pone.0190218

**Published:** 2017-12-27

**Authors:** John-Ih Lee, Michael Gliem, Gebke Gerdes, Bernd Turowski, Marius Kaschner, Bastian Kraus, Hans-Peter Hartung, Sebastian Jander

**Affiliations:** 1 Department of Neurology, Heinrich-Heine-University, Medical Faculty, Duesseldorf, Germany; 2 Department of Diagnostic and Interventional Radiology, Heinrich-Heine-University, Medical Faculty, Duesseldorf, Germany; University Hospital-Eppendorf, GERMANY

## Abstract

**Background:**

In a proportion of stroke patients with acute large vessel occlusion permanent stent implantation is mandatory to achieve successful recanalization. The optimum platelet inhibition strategy after such emergency stenting is unknown. We therefore analyzed the outcome of early glycoprotein (gp) IIb/IIIa inhibitor treatment after emergency stenting in acute stroke.

**Methods:**

Sixty patients with emergency stenting were identified in our stroke unit registry from 12/2010-06/2014 and analyzed retrospectively. All patients were bridged intravenously with the gpIIb/IIIa antagonist tirofiban immediately after the acute procedure until switching to oral aspirin and clopidogrel was performed. For comparison we studied 135 patients with M1 occlusion undergoing thrombectomy without stent implantation or tirofiban treatment in a propensity score-adjusted analysis.

**Results:**

In the acute stenting group receiving tirofiban complications with 6 deaths during the hospital stay (10%), 2 reinfarctions (3%), 12 intracerebral hemorrhages (ICH; 20%) and 5 symptomatic ICH (8%) occurred. Thirty-seven patients (62%) reached a moderate outcome of mRS 0–3 after 90 days. In the thrombectomy group without tirofiban administration the rate of deaths within hospital stay, the rate of ICH and outcome at day 90 were not different.

**Conclusion:**

In our retrospective study acute stenting with subsequent gpIIb/IIIa inhibition was not associated with an increased risk of ICH or in-hospital death.

## Introduction

In stroke patients with acute large vessel occlusion and stenoocclusive atherosclerotic lesion stent implantation in addition to clot retrieval may be necessary to access downstream embolic occlusions or ensure lasting recanalization. The optimum platelet inhibition strategy in such situations is currently unclear. Glycoprotein (gp) IIb/IIIa inhibitors such as tirofiban can be administered intravenously, exhibit fast onset of action, and the effect subsides within a few hours after discontinuing infusion. Despite reports of an increased risk of secondary intracerebral hemorrhage (ICH) after ischemic stroke [1–3], tirofiban is still used in acute stroke with emergency stent implantation, mostly as bridging medication until dual platelet inhibition with oral clopidogrel and aspirin is effective. The goal of our study was to analyze safety and outcome of tirofiban treatment following emergency stenting in acute stroke patients.

## Methods

### Study population

As approved by the local ethics committee [Ethikkomission der Medizinischen Fakultät der Heinrich-Heine-Universität Düsseldorf (#4743R)], routine medical care data of all patients treated for ischemic stroke in the Stroke unit of the Department of Neurology, Heinrich-Heine-University, Duesseldorf from 12/2010–06/2014, were collected in an anonymized and pseudonymized manner (n = 2600) and analyzed retrospectively. For observational retrospective analysis a separate written informed consent was not required by the local ethics committee.

We identified 60 patients with acute ischemic stroke in the anterior circulation, who received acute stenting of extra- and/or intracranial arteries in addition to endovascular thrombectomy in the same intervention with or without preceding i.v. thrombolysis. All these patients, except for one patient with early ICA stent occlusion during intervention, were treated with the gpIIb/IIIa antagonist tirofiban (1.250 mg bolus during intervention followed by a continuous infusion of 0.1μg/kg body weight/minute) from time of acute stenting until a switch to aspirin (500 mg loading dose i.v. following 100 mg once daily orally from the next day on) and clopidogrel (600 mg loading dose following 75 mg once daily orally from the next day on) was performed, mostly within 12–24 hours, with 12 hours overlap. For comparison we analyzed 135 patients with ischemic stroke who received endovascular thrombectomy of the middle cerebral artery (M1 segment) occlusion without stent implantation or tirofiban treatment.

### Diagnostic tools

Imaging was performed with a 3-T or 1.5-T MR scanner (MRI: T2*, DWI, ADC, FLAIR, TOF) or contrast enhanced CT (2 mm slices including 5 mm reconstructions, 0.75 mm slices for CT-angiography). Alberta stroke program early CT score (ASPECTS) [4] were obtained by two neuroradiologists blinded for therapeutic procedures and outcome on initial imaging (pretreatment) and follow up CT Scan 12–24 hours after intervention (posttreatment).

### Outcome assessment

Clinical outcome was assessed by trained physicians employing modified Ranking Scale (mRS) at hospital discharge [5], and after 90 days with a standardized telephone questionnaire [6]. Modified Ranking Scale (mRS) ≤3 was considered as moderate and mRS >3 as poor outcome. Complications including death during the hospital stay, reinfarction, any ICH, and symptomatic ICH were documented and analyzed. Any ICH was defined as any type of hemorrhagic transformation including hemorrhagic infarction and parenchymal hematoma [7]. Symptomatic intracerebral hemorrhage (sICH) was defined according to the ECASS 3 definition (any hemorrhage with neurologic deterioration as indicated by an NIHSS score that was higher by ≥4 points than the value at baseline or the lowest value in the first 7 days, or any hemorrhage leading to death; in addition, the hemorrhage must have been identified as the predominant cause of the neurologic deterioration) [8]. All observed symptomatic intracranial bleedings were intracerebral.

### Statistical analysis

SPSS Statistics 20 was used for statistical analysis. Dichotomized parameters were compared by use of Chi-Square test while continuous data were analyzed with Mann-Whitney-*U* test (Table 1). Clinical outcomes and complications were compared by propensity score-adjusted analysis (Table 2) adjusting unequally distributed baseline parameters (age, gender, NIHSS, diabetes mellitus, atrial fibrillation, smoking, TICI Score, pre- and posttreatment ASPECTS) into a propensity score by logistic regression analysis. P<0.05 was assumed statistically significant.

**Table 1:** Baseline parameters.

Baseline Characteristics	Acute Stenting with Tirofiban n = 60	Acute MCA thrombectomy without Tirofiban n = 135	p Value
Male gender	38 (63%)	55 (41%)	**p = 0.004**
Median age in years (IQR)	71 (61–77)	76 (69–82)	**p = 0.004**
Median NIHSS Score (IQR)	11 (6–16)	15 (11–18)	**p<0.001**
Arterial hypertension	48 (80%)	116 (86%)	p = 0.296
Diabetes mellitus	7 (12%)	38 (28%)	**p = 0.012**
Atrial fibrillation	15 (25%)	74 (55%)	**p<0.001**
Hyperlipidemia	36 (60%)	61 (45%)	p = 0.056
Smoking	20 (33%)	12 (9%)	**p<0.001**
Previous stroke	6 (10%)	13 (10%)	p = 0.936
Coronary heart disease	7 (12%)	23 (17%)	p = 0.337
Bridging i.v. thrombolysis	48 (80%)	116 (86%)	p = 0.296
Time from symptom onset to i.v. thrombolysis in minutes (IQR)	88 (63–128)	82 (62–120)	p = 0.712
Time from symptom onset to groin puncture in minutes (IQR)	175 (139–238)	183 (143–294)	p = 0.325
Time from symptom onset to recanalization in minutes(IQR)	263 (217–332)	264 (207–356)	p = 0.646
Median pretreatment ASPECTS (IQR)	10 (9.75–10)	10 (9–10)	**p = 0.042**
Median posttreatment ASPECTS (IQR)	8 (7–9)	7 (5–8)	**p = 0.007**
TICI Score ≥ 2b	54 (90%)	103 (76%)	**p = 0.026**

**Table 2:** Outcome analysis.

	Acute Stenting with Tirofiban n = 60	Acute MCA thrombectomy without Tirofiban n = 135	After propensity score adjustment, OR with CI and p-value
Discharge mRS 0–3	32 (53%)	60 (44%)	**OR 3.164 [1.306–7.668]; p = 0.011**
90 days mRS 0–3	37 (62%)	66 (49%)	OR 2.129 [0.930–4.872]; p = 0.074
Reinfarction	2 (3%)	1 (1%)	n.a.*
Any ICH	12 (20%)	27 (20%)	OR 0.863 [0.370–2.013]; p = 0.734
Symptomatic ICH	5 (8%)	8 (6%)	OR 1.706 [0.388–7.505]; p = 0.480
Death within hospital stay	6 (10%)	14 (10%)	OR 3.292 [0.797–13.597]; p = 0.100

* numbers to small

## Results

All patients undergoing acute stenting of extra- and/or intracranial arteries of the anterior circulation received tirofiban treatment except for one patient with early ICA stent occlusion during intervention. This patient remained in the stenting group for baseline parameter and outcome analysis. Stent implantation was performed into the extracranial ICA (n = 47), the MCA (n = 8), the extracranial and intracranial ICA (n = 1) as well as the extracranial ICA and MCA (n = 4). Twelve (20%) patients had an isolated occlusion of the extracranial internal carotid artery with patent M1 or carotid T, 22 (37%) had tandem occlusions including ICA and M1 or carotid T, while 26 (43%) had intracranial (M1, M2, carotid T) occlusions.

For comparison, we identified 135 patients who received endovascular thrombectomy of the M1 without stent implantation or tirofiban medication. Pre- and posttreatment ASPECTS was significantly higher in the stenting group. Recanalization with TICI Score ≥ 2b was achieved significantly more frequent in the stenting group.

To account for dysbalance in these and other baseline parameters (see Table 1), outcome parameters were assessed by propensity score-adjusted analysis. Of note, time from symptom onset to i.v. thrombolysis, groin puncture and recanalization did not significantly differ between the groups (Table 1). Clinically relevant major complications (reinfarction, any ICH and sICH according to ECASS 3 definition), death within hospital stay and mRS after 90 days did not differ between both groups (Table 2). The mRS at discharge was significantly better in the thrombectomy group without stenting, while there was no significant mRS difference at day 90 anymore. Stratification into extra- und intracranial stenting site (47 extracranial and 1 extra-and intracranial ICA stenting patients compared to 135 acute MCA thrombectomy patients, intracranial stenting patients (8 M1 + 4 ICA and M1) compared to 135 acute MCA thrombectomy patients, ICA stenting patients (47 extracranial and 1 extra-and intracranial) compared to intracranial stenting patients (8 M1 + 4 ICA and M1)) did not reveal any difference regarding the outcome parameters (data not shown).

## Discussion

Optimum platelet inhibition strategies following emergency stent implantation in acute ischemic stroke are controversial. Preclinical experimental data in mice [3] showed an increased rate of intracerebral hemorrhage and death without reducing stroke volume following gpIIb/IIIa blockade. In patients, aspirin, clopidogrel, gpIIa/IIIb antagonists [9–11] and combinations are used with variable results regarding gpIIa/IIIb antagonist-associated complications [1,2,9–11]. But often in these studies an appropriate comparison group was lacking [9–11]. Furthermore, in a study detecting an increased risk of fatal ICH and poor outcome [1] tirofiban was used if endothelial damage was suspected, e.g. because of multiple thrombectomy passages during the recanalization procedure [1] which might increase the bleeding risk.

Due to a favorable safety profile in the previous SATIS Trial [12] the gpIIb/IIIa antagonist tirofiban is still used at our stroke center as bridging platelet inhibition in the specific situation of emergency stenting in acute stroke. In our present study we analyzed the safety and outcome of tirofiban treatment if used as bridging platelet inhibition in acute stroke due to large vessel occlusion requiring emergency stent implantation. For comparison we studied acute thrombectomy patients with large vessel occlusion but without stent implantation or tirofiban treatment. In propensity score-adjusted analysis acute stenting followed by tirofiban bridging was not associated with a higher rate of major complications, in particular ICH or death, or a worse stroke outcome after 90 days. While we observed better mRS scores at discharge in the thrombectomy group, outcome was similar at day 90 after stroke. In addition, the rate of sICH was within the range reported from the MR CLEAN trial of thrombectomy [13] in intracranial large vessel occlusion. Thus, tirofiban administration was not associated with an excess of clinically relevant intracerebral bleeding or worse overall outcome.

Limitations of our study are the retrospective design, small sample size, and lack of randomization. Therefore, controlled trials are needed to establish platelet inhibition strategies in acute stroke patients treated by emergency stent implantation.
